# Behind the data: Reflections from conducting school-based suicide prevention research with young people in the United Kingdom

**DOI:** 10.1371/journal.pmen.0000476

**Published:** 2025-10-30

**Authors:** Molly McCarthy, Sio Wynne, Emma Ashworth, Olivia Hendriks, Pooja Saini

**Affiliations:** School of Psychology, Liverpool John Moores Univeristy, Liverpool, United Kingdom; PLOS: Public Library of Science, UNITED KINGDOM OF GREAT BRITAIN AND NORTHERN IRELAND

## Abstract

Suicide is the leading cause of death among children and young people under 35 in the United Kingdom (UK), and suicide rates in this group are rising. Schools are considered an appropriate and logical setting for suicide prevention activities, with universal access to a wide range of young people. However, schools are complex settings, meaning research projects often encounter a number of logistical, engagement, and implementation challenges. This commentary presents learnings from a regionally based feasibility trial of an integrated response to suicide risk among UK secondary schools. We explore the barriers and challenges to engaging and conducting research on suicide and self-harm prevention with young people and offer recommendations for researchers.

## Introduction

Suicide is the leading cause of death among children and young people under 35 in the United Kingdom (UK), and rates have been shown to increase by 7–9% per year since 2010 [1]. Adolescents (aged 10–19) are considered one of the most vulnerable groups affected by suicide, with physical and mental changes, as well as the life events during that stage, significantly increasing risk [2]. Adolescents with suicidal ideation face a significant number of barriers to accessing mental health services, with only 28% receiving any professional intervention [3]. Beyond structural barriers, individuals face additional fears of hospitalisation and stigma, impacting on help-seeking behaviours [4,5]. Therefore, there is an urgent need to provide effective and appropriate suicide prevention support for this vulnerable population.

Yet, research in this area, with this population, can be difficult to implement for a number of reasons. Ethical bodies are often concerned with the impact of talking about suicide and self-harm on young people, with concerns that it may increase their risk; for example, Lakeman and FizGerald [6] reported that 65% of respondents thought that suicidality may be “reinforced” through bring attention to suicidal thoughts and feelings. However, there is no evidence to suggest iatrogenic effects of suicide prevention programmes, and the overall consensus in the literature promotes and encourages young people’s involvement in suicide prevention research [7]. Engaging young people not only ensure their voices are heard and represented, but also helps tailor interventions to their specific cognitive, emotional and social needs, which differ significantly from those of adults [7]. Moreover, involving young people in the design and implementation of research can enhanced the relevance and acceptability of interventions [8]. This is particularly crucial given the importance of early intervention for suicidal thoughts and self-harm.

The majority of young people spend significant amounts of time in school; thus, schools are considered an appropriate and logical setting for youth suicide prevention activities, providing universal access to a wide range of young people [9,10]. Compared to primary care settings (e.g., GPs), school-based research can overcome barriers such as location and time, and has been shown to support relationships between both peers and teachers [11,12]. Furthermore, school-based interventions may also benefit young people whose access to services are limited, for example ethnic or religious minorities and those from lower socio-economic status [13–15]. Young people have also found school-based interventions to be more accessible than community-based programmes [16], providing them with access to education, and/or support that they would otherwise lack access to [9,10].

Despite the noted benefits of conducting suicide prevention work with young people, school settings present unique challenges. Often the key barrier to research in this setting is logistics, with staff time being a particular challenge [17]. Schools face multiple competing demands and external pressures, particularly at certain times of the year (i.e., examination periods, OFSTED inspections) [18]. Staff are also often required to find time to complete research-related administrative tasks, such as send consent forms to parents/carers or book classrooms, which can be difficult in addition to their normal day-to-day teaching and tasks [19]. In the UK, schools are also continually measured on their ability to meet specific academic outcomes and cover an extensive curriculum [20], which can sometimes make it more difficult to find space in the calendar for research-related activities. The success of research is, therefore, dependent on a wide range of factors and ability to manage competing demands. It is clear that strategies and methods to mitigate these are needed to lessen the burden on schools, and support efficient research efforts [19].

The Multimodal Approach to Preventing Suicide in Schools (MAPSS) project is a suicide prevention programme for Year 10 pupils that aims to reduce suicide risk and increase help-seeking among young people. It consists of three parts:

(1) a universal suicide prevention lesson for all pupils,
(2) risk screening using the Suicidal Ideation Attributes Scale (SIDAS) to identify those at risk, and
(3) an online cognitive behavioural therapy (CBT; ‘ReframeIT’) for those deemed to be at high risk for suicidal ideation.

For more information see Ashworth et al. [21]. MAPSS was originally developed in Australia by Orygen at the University of Melbourne and has been shown to be feasible and acceptable in Australian schools [22]. As cultural transferability cannot be assumed, MAPSS is currently being implemented and tested in an ongoing 2-year regionally based feasibility randomised controlled trial across six secondary schools in the United Kingdom.^21^

In this paper, we present our reflections from engaging with schools throughout MAPSS and offer recommendations for other researchers in similar fields looking to work with schools. Our aim is to share our learnings from setting up and conducing this research within school settings, to help others avoid and/or mitigate some of the challenges we faced and share techniques and strategies which we found useful. While some of the approaches to research discussed here will be specific to the English school context and environment, and may be amplified due to the nature of the topic, the learning from this project illustrates many of the key challenges that arise when conducting mental health research in schools globally.

## Key Learnings

The following section highlights some of the key challenges and learnings we have experienced as part of the MAPSS UK-based feasibility trial. We share some common challenges we faced, as well as what worked well for supporting programme implementation and research. Table 1 provides a summary of the key challenges and solutions we faced.

**Table 1:** Summary of challenges and solutions.

Category	Challenge	Solution
Building Connections and Facilitating Staff Engagement	Communicating with staff who have very little time for research administration. Involving the right person.	Break the research down into clear phases and steps. Summarise main point at the end of an email. Send follow-up emails. Work with multiple members of staff, if possible.
Managing Ongoing Pastoral Requirements and Responding to Risk	Ensuring risk is dealt with in a timely manner and school safeguarding procedures are followed. Sustaining school engagement.	Clear communication of risk, explaining survey measures to school staff. Providing supervision for school staff managing risk. Having multiple contacts for the school.
Staff Confidence and Parental Buy-In	Staff fearful of saying the “wrong thing”. Poor parental engagement.	Providing training for both staff and parents. Holding sessions at times convenient for staff/ parents that do not add to their workload (i.e., training days, GCSE information evenings).
Logistical Challenges When Conducing Quantitative and Qualitative Research	Accessing IT/ computers for surveys. School phone policy. WIFI and signal difficulties. Staff capacity during visits.	Be aware and up to date with school calendar (i.e., half term dates). Provide additional short and concise documents to support school staff who may have no knowledge of project and parents. Be flexible in your availability to support school schedules. Check IT availability and school policies in advance.
Importance of PPI	Who to include? How do you recruit individuals to participate in these groups?	Use local networks. Provide payment (i.e., vouchers) for time so members feel valued.

### 1. Building connections and facilitating staff engagement

Building relationships with schools is essential to ensure staff are aware of the project aims, what is involved, and the required commitment, to facilitate effective implementation of the programme. Members of staff involved in the project will also have differing levels of experience with research and suicide prevention, with some having never participated in research previously and/or never spoke or discussed suicide prevention with young people [19]. We found school staff are more willing to engage and support the project needs if they understand its importance and purpose, and can see the direct benefits of the work they are putting in for their students. For example, in MAPSS, school staff saw a direct positive of the programme through the identification of young people at risk, who have previously been ‘under the radar’:

“*I didn’t even know the young people that had come up like I’d never even seen them before, like I had to actually like, search their name and look for their picture, so that was quite interesting.”* (School Staff 1).

It is also important to highlight from the beginning the aims of the project, key stages, and timelines. Through email communication, this can be reiterated, as well as consistently expressing thanks to schools for their time and support.

Good communication with schools is crucial. In our experience, regular and consistent email contact worked the best, allowing staff to respond at a time that was suitable for them and/or follow up with young people or other staff members. We found personalised communication and building individual rapport more effective than generic emails, helping researchers build a relationship with staff. School staff who took part in the MAPSS programme noted the value of having regular reminder emails and follow-ups:

“*Everyone’s been really good in terms of like communication. I just think sometimes it’s hard, like I have so many different emails and things and stuff, but I feel like if I’ve ever needed to contact anyone, they’ve always contacted me back straight away. I don’t think I’ve always been the same and even just like the reminders, I’m always a fan of a constant reminder*.” (School Staff 1).

Staff engagement is essential to the success of any school-based trial. With that, it is important to understand staff roles, their responsibilities, and extra-curricular commitments (e.g., lunch duty). In many cases, staff members were not relieved of their regular responsibilities to support the MAPSS project, leading to additional work outside of standard hours, such as calling parents in the evenings. Often, the responsibility fell on a single staff member, increasing staff stress and resulting in limited capacity to implement the programme effectively. This highlighted the need for schools to allocate sufficient resources and staffing when engaging with similar initiatives. Again, communication is essential to ease the burden on staff by clearly highlighting what is needed in advance, as well as reiterating the importance of the project and gratitude for their support. If it is possible to have multiple staff involved in the research, this should be encouraged from the start.

Additionally, hierarchies often exist within school settings, which sometimes result in activities having to be authorised by senior staff members (i.e., Headteachers). Similar to other research [19], we found difficulties both when junior staff were trying to proceed without senior involvement, and when a senior staff member committed to the research without the buy-in from classroom teachers/safeguarding team who would be required to be involved. Here, it is important to have multiple school contacts, with an understanding of who is responsible for what within the school environment. Feedback from school staff involved with MAPSS similarly re-emphasised how useful having email reminders and follow-ups were, purely for the purpose of being able to send this to other senior staff to get approval:

“*The emails and explanations you sent was really helpful because then I can forward it to the safeguarding lead and go ‘look, I really need to respond, we really need to book something in’…”* (School Staff 4).

Implementing a whole-school approach is also important, particularly when the staff members initially engaged are not the ones directly involved in day-to-day implementation. While securing individual staff buy-in is crucial, raising awareness across the entire school community is equally as important to ensure smooth delivery. We found that when administering questionnaires during form time, some form teachers were unaware of the project’s aims, leading to uncertainty and increased stress as they were unable to confidently answer students’ questions. This highlighted the need for clear, accessible communication across all levels of staff. To address this, we developed a concise, one-page summary document for form teachers, providing an overview of the project’s purpose and key objectives, along with answers to frequently asked questions from students. We also ensured two researchers were present within the school during completion of the questionnaires. We found this not only reduced attrition but also provided school staff with reassurance when difficult questions were brought up.

Through MAPSS, we also found it was essential to be able to address any concerns raised by staff in a timely, effective, and empathetic manner. This is particularly key when dealing with sensitive topics, such as young people’s mental health and suicidal thoughts. We found staff were sometimes worried about how to approach these conversations with young people in fear of saying the wrong thing, highlighting the need for clear guidance and support. To help with this, PAPYRUS Preventing Young Suicides charity delivered their SP-OT training, providing knowledge to school staff on how to talk about suicide, as well as spot the warning signs. Additionally, concerns around increased workload for school staff must be acknowledged from the outset by providing clear and transparent information on what the project entails and ensuring expectations are manageable. Establishing a Memorandum of Understanding (MoU) with schools at the beginning of the project can help to clarify responsibilities, reinforce commitments, and create a structured framework that supports staff to understand the phases of the research. By actively addressing these concerns, we can foster a more supportive and effective research environment, ultimately benefiting staff, students, and researchers.

### 2. Managing ongoing pastoral requirements and responding to risk

Managing the ongoing pastoral requirements for suicide prevention work with young people is essential to ensure the safety, wellbeing and ethical involvement of all participants – especially given the sensitive nature of the topic.

The safeguarding of young people within suicide prevention research is imperative to ensure safe, ethical research. As part of MAPSS, young people filled out a survey which included direct questions about suicidal thoughts/ self-harm. Researchers were then responsible for feeding this information back to schools, with schools then required to follow-up with students within 24 hours, in accordance with their normal safeguarding procedures. Again, open communication between researchers and the school was essential to ensure the list of students had been received and acknowledged. To reduce stress for school staff, we arrange surveys to take place at the start of the week, and avoided conducting surveys on a Friday or before a school holiday, where the young people at risk may not have been followed up immediately.

The perception of risk in suicide prevention research was not always well understood by schools, often generating concern among staff and leadership. Introducing discussions around suicide and self-harm can heighten anxieties about student safety and the school’s role and responsibilities. This was particularly evident following the risk screening as schools were surpirsied with the number of young people identified:

“*I wasn’t expecting such a large number of our young people to come back flagged up high risk…And obviously you’ve got that that risk hanging over you in terms of like how much time you have to speak to the young people and phone parents.”* (School Staff 1).

Managing this tension required clear communication about the purpose, safety protocols and long-term benefits of such research. Schools were provided with detailed information on how students were identified as at-riak from the surveys and explanations of the cut-offs to deem ‘medium’ and ‘high risk’. It was also highlighted to schools when young people had indicted a suicide plan. To further support staff during this process, one-to-one supervision with a trained CBT therapist was offered. This gave staff a confidential space to reflect on their experiences and develop their confidence in responding to high-risk sutiations.

*“I found the supervision really helpful. Even like last week, we spoke specifically about those young people who’d flagged as high risk, and [X] was able to send me over like a booklet and some different safety plans. So, I suppose for me it just kind of put my mind at ease a little bit.”* (School Staff 1).

There were also incidences where school engagement declined during the course of the programme, which raised concerns for the research team, particularly in cases where we were aware that some young people were experiencing suicidal thoughts but we did not know what support, if any, they had received. Sustained school involvement is crucial for ensuring appropriate support structures remain in place for vulnerable students. A lapse in school engagement can leave at-risk students without timely follow-up or intervention, which was particularly problematic when risk had been identified. We found school engagement sometimes dipped prior to the start of ReframeIT, mostly impacted by staff sickness, staff turnover or competing school priorities. Thus, maintaining ongoing communication with follow-up emails and calls helped to ensure safety protocols are being followed, as well as ensuring researchers have multiple contacts for the school.

Building a supportive and open research environment was also essential to manage the ongoing pastoral requirements, by fostering strong relationships with school staff, but also in establishing trust and transparency with the young people involved. For example, during the administration of surveys, two researchers were present in-person to provide reassurance, clarify any questions and ensure the surveys ran smoothly. We received questions about confidentiality and use of their data, but also the broader purpose and value of suicide prevention research. For young people, this process was also seen as an opportunity to reach out for support in school, allowing students to “*have a voice*”, “*without feeling judged*” or *“pressured into talking”* (Young People 6; 8; Focus Group).These interactions highlighted the importance of maintaining a visible, supportive presence throughout the research process both for young people and school staff.

### 3. Staff Confidence and Parental Buy-In

Staff confidence is also another critical aspect when conducting suicide prevention research and implementing suicide prevention programmes within school settings. Although pastoral and/or safeguarding staff generally have mental health training (albeit not always suicide prevention focused), other staff, such as form teachers, often do not receive such training, despite being the young person’s “safe” teacher within the school. This can contribute to increased stress and emotional burden among staff, feeling unprepared to handle conversations with fear of saying the “wrong thing” [23 ]. As highlighted in previous studies [24,25], school staff often report a sense of responsibility without feeling adequately supported, which can lead to resistance against engaging with suicide-related programmes and research.

Moreover, parental buy-in is equally as important to ensure ethical and effective suicide prevention research with young people. Parents and caregivers often serve as gatekeepers to participation, and their support can influence both consent rates as well as broader acceptance of the research in the school community [26]. Parents expressed concern about exposing their child to discussions about suicide and self-harm, fearing that it would cause distress and increase risk. Addressing these concerns requires transparent, accessible communication through information sheets, consent forms and opportunities to ask questions. In addition to this, we offered schools PAPYRUS SPOT (Suicide Prevention Training for Teachers) and SPARK (Suicide Prevention Awareness Raising for Parents) training, which Uptake in parent training was mixed, but proved most effective when scheduled alongside existing school events (e.g., GCSE information evening).

Similarly, for staff training, it was important to engage individuals across different roles within the schools, ensuring form teachers, subject teaching staff, and pastoral staff all had the opportunity to participate and maximise both the reach and impact across the whole school environment.

“*It was powerful because we had curriculum leads going back to their departments and talking about this training and that resonates a little bit more with staff rather than me [safeguard lead] talking about it… instead, it’s a random maths teacher talking about suicide prevention and I think that was really powerful*.” (School Staff 8).

### 4. Logistical challenges when conducing research

Although research in school settings has always required significant effort and persistence, there is strong consensus across the literature and practice that over that the past two decades this has become increasingly difficult [27]. Logical challenges are one of the main issues researchers face when conducting work within school settings.

School staff often have complex roles, balancing teaching with additional responsibilities such as pastoral care, extracurricular tasks, and administrative duties [28]. This makes it challenging for researchers to find time to engage with staff, support their understanding of the programme, and ensure they feel confident in their role within the research process. Similarly, staff may struggle to find the time to implement necessary tasks, such as distributing consent forms to parents, amidst their existing workload and full teaching schedule. Here, good planning is essential – not only in terms of supporting engagement opportunities (phone calls, Teams meetings), but also in aligning research activities with the realities of the school environment. Understanding the school calendar, including term dates, exam periods and holidays, is crucial to avoid placing additional strain on staff during particularly busy times. For example, during ReframeIT we had to work flexibly with schools to ensure the 8-weeks of content could fit into the school term and pause access to modules if a half-term fell during the time. This was more problematic during the summer term, when schools were managaing multiple competing demands such as GCSE mock exams and a range of extracurriocualr activities. For example, “*We’ve had a big issue with space computer space because of the time of year it is, because we’re in GCSE season, we also have a lot of trips this time of year…So, in one sense it would have been better to do the MAPSS programme earlier in the academic year.*” (School Staff 1). These pressures often meant reduced staff avaliabiliy and limited capacity to enage fully with the prpgramme. To accommodate this, we offered adapted scedhuling options, allowed for breaks in delivery, and maintained regular communication with schools.

Conducting quantitative research in schools also presents several logistical challenges, particularly when requiring opt-out consent from parents. While opt-out consent can increase student participation rates [29], ensuring that all parents are adequately informed and given the opportunity to withdraw their child requires careful planning and clear communication. Schools often serve as the connection between researchers and parents for distributing information, meaning that researchers must rely on staff to send consent forms home, track responses, and manage any withdrawals [30]. However, competing demands on staff time and varying levels of engagement can lead to delays or inconsistencies in distribution. To address this, considerable effort was made to ensure fully informed consent by providing clear, accessible materials to parents, including information sheets and a frequently asked questions document.

Finally, the logistics of the school setting also provided certain challenges for the quantitative data collection aspect of MAPSS. Issues, such as unreliable Wi-Fi, room booking, and finding time within a busy school calendar, hindered the data collection process. If research involves the use of mobile phones (i.e., for completing surveys), it is also important to check the schools phone policy for the young people. This is particularly important following pressures from the UK government for a ban on phones within schools [31]. For example, in one school students were required to lock their phones in pouches for the whole school day. This meant measures needed to be put in place to unlock pouches and allow young people access to their phones to complete the required survey. Additionally, some students opted to not bring their phone to school due to policy, thus, it was important for schools to inform students in advance that phones may be needed. In cases where students did not have access to a phone, schools were required to provide laptops or book computer spaces. Again, obtaining whole school buy-in is crucial to ensure the needs of both the students and staff are considered.

### 5. Importance of co-design

The involvement of young people within suicide prevention research is an evolving field, with increasing research indicating the benefits of involvement [7]. Involving young people can improve the relevance of the research being conducted, as well as the transferability of findings [32]. Involvement also gives an opportunity for young people to have their voices heard, get experience of supporting a research programme [33], and positively influence outcomes, showing a direct correlation with reduced stigma and discrimination [34]. Challenges, however, arise commonly with ethics committees, concerned with whether involving young people with lived experience of suicidal thoughts and/or behaviours will result in adverse effects [35]. However, this has been shown to not be the case – Blades et al.’s [36] meta-analysis reported participation in suicide research may actually offer small but significant benefits to participants, with adolescents showing nearly twice as large a reduction in suicidal thoughts pre- to post-exposure, compared with adults. It is important to highlight this information to ethics committees and demonstrate the benefits of young people involvement [32]. Equally as important is ensuring young people themselves are fully informed and given the opportunity to provide their own assent or dissent. Even when parental consent has been granted, it is essential that the young person retains the right to decline participation if they choose. This was achieved from age-appropriate videos, assembly presentations and in-person visits from the researchers to allow students to ask any questions.

As part of the refinement of the MAPSS programme following the pilot study, three informational videos were co-produced with young people to provide additional information about the aspects of MAPSS (e.g., surveys, ReframeIT). Feedback from the pilot indicated that some participants felt they did not have enough information about the programme, thus, in response to this the research team collaborated with young people to create short and accessible videos to explain MAPSS. To further enhance relatability, voiceovers were provided by the young people themselves. These videos were then used during school assemblies prior to the start of data collection, to ensure all students received clear and consistent information in an engaging format.

Researchers may also be unsure of how to access/recruit young people for advisory involvement. Our suggestion is to involve local partners and charities supporting young people to allow for young voices to be heard. For example, Merseyside Youth Association (MYA) in Liverpool were heavily involved in the recruitment of young people for the MAPSS advisory group. MYA is a charity providing support and opportunities for young people, and involves a range of groups (e.g., MYA RAISE to support mental health) [37]. Collaboration with local partners early in the project allowed for strong connections to be established, rapport building, and encouraged active involvement. We also held the group sessions on their site, to allow young people to feel more comfortable in the environment and setting, with other young people and staff who they already knew and trusted. In line with the National Institute for Health Research (NIHR) PPI guidelines, young people were reimbursed for their time in the form of vouchers [38].

The young people’s advisory group (YPAG) met tri-annually, with each session running for about two hours. The sessions involved a catch-up/introduction (often with biscuits), project progress update, then a main activity which differed each time, for instance qualitative interview feedback, which involved young people reading a transcript and sharing their thoughts. Another session was spent co-developing additional documents to support school staff. This was due to staff feedback on how young people were being told they were identified as ‘at-risk’ as part of the screening. Having discussed this at one YPAG meeting, the team decided it was important to co-develop, with the YPAG, a guidance document for school staff on things to say/not say to young people, and how to inform them about the opportunity to participate in ReframeIT. For example, see Box 1 below for suggestions:

**Box 1. Section taken from co-developed guidance document with YPAG for school staff.**

Students may appreciate you as staff being able to answer any questions about MAPSS and Reframe-IT. We have included some information below, but if you have any further questions please do contact us at MAPSS@ljmu.ac.uk.

**Suggestions from Young People:**

- It is the student’s choice whether to take part in Reframe-IT or not.
- Try to offer students as much choice as possible regarding how, when, where, and with who they complete Reframe-IT.
- Students may like a reason to tell teachers or peers why they are leaving a lesson for Reframe-IT. Some may have an idea of what they’d like to say; if not, an example, could be as simple as saying they’ve been randomly chosen to take part in an extra element for MAPSS.
- If possible, consider allowing students to nominate a ‘safe teacher’ who can support them while completing Reframe-IT.
- Each module should take around 20 minutes to complete, but students may find it helpful to miss a whole lesson to take some time to decompress afterwards.

As well as active involvement from a young person advisory group (YPAG), an adult advisory group also informed on the project development. Again, the group met tri-annually and involved individuals parents of children with lived experience and adults with experience as a child. A particularly important outcome from the adult advisory group was the co-development of a parent frequently asked questions (FAQ) document. This was developed in response to the common misconceptions and myths about suicide often being cited by parents when opting their young person out of MAPSS. The group spoke about how parents may not actually have the time to sit and read the full participant information sheet, highlighting the value of the research, and parents may benefit from a short concise information document. As such, the FAQ document was developed to provide clear, accessible information to highlight the facts about youth suicide, for example: “Talking about suicide provides the opportunity for communication and prevention, may encourage help-seeking, and there is no evidence to suggest that talking about suicide increases risk.”.

## Conclusions

This commentary highlights the challenges many researchers face when conducting school-based research. Some of the noted challenges may also be amplified for those exploring mental health, particularly suicide prevention research. Building connections with staff from the start is important to ensure staff engagement and expectations are met. Clearly summarising each stage of the research, working with multiple staff members, as well as sending regular follow-up emails can help mitigate concerns with engagement. As discussed throughout, schools provide a highly important setting for research, providing access to a wide range of young people, for whom access to services and support may be limited. Nonetheless, logistical challenges can make implementing research programmes within schools difficult. It is important to consider IT/computer access, room bookings, school calendar dates (e.g., half-term), and to be flexible in your availability to support school schedules. Finally, including young people’s voices is important to consider; connect with local partners and charities to support this and ensure young voices are heard. In summary, our findings from MAPSS highlight the importance of school-based research, yet demonstrate the need for careful planning and consideration when involving schools in research.
